# A New Route for Preparation of Hydrophobic Silica Nanoparticles Using a Mixture of Poly(dimethylsiloxane) and Diethyl Carbonate

**DOI:** 10.3390/polym10020116

**Published:** 2018-01-26

**Authors:** Iryna Protsak, Evgeniy Pakhlov, Valentyn Tertykh, Zi-Chun Le, Wen Dong

**Affiliations:** 1 College of Science, Zhejiang University of Technology, Hangzhou 310023, China; ips@zjut.edu.cn; 2 Chuiko Institute of Surface Chemistry of National Academy of Sciences of Ukraine, 03164 Kyiv, Ukraine; pakhlov.e.m@gmail.com (E.P.); tertykh@voliacable.com (V.T.); 3 College of Environment, Zhejiang University of Technology, Hangzhou 310023, China; dongwen@zjut.edu.cn

**Keywords:** poly(dimethylsiloxanes), surface modification, nanosilica, diethyl carbonate, carbon content, morphology, coatings, fillers

## Abstract

Organosilicon layers chemically anchored on silica surfaces show high carbon content, good thermal and chemical stability and find numerous applications as fillers in polymer systems, thickeners in dispersing media, and as the stationary phases and carriers in chromatography. Methyl-terminated poly(dimethylsiloxanes) (PDMSs) are typically considered to be inert and not suitable for surface modification because of the absence of readily hydrolyzable groups. Therefore, in this paper, we report a new approach for surface modification of silica (SiO_2_) nanoparticles with poly(dimethylsiloxanes) with different lengths of polymer chains (PDMS-20, PDMS-50, PDMS-100) in the presence of diethyl carbonate (DEC) as initiator of siloxane bond splitting. Infrared spectroscopy (IR), elemental analysis (CHN), transmission electron microscopy (TEM), atomic force microscopy (AFM), rotational viscosity and contact angle of wetting were employed for the characterization of the raw fumed silica and modified silica nanoparticles. Elemental analysis data revealed that the carbon content in the grafted layer is higher than 8 wt % for all modified silicas, but it decreases significantly after sample treatment in polar media for silicas which were modified using neat PDMS. The IR spectroscopy data indicated full involvement of free silanol groups in the chemisorption process at a relatively low temperature (220 °C) for all resulting samples. The contact angle studies confirmed hydrophobic surface properties of the obtained materials. The rheology results illustrated that fumed silica modified with mixtures of PDMS-x/DEC exhibited thixotropic behavior in industrial oil (I-40A), and exhibited a fully reversible nanostructure and shorter structure recovery time than nanosilicas modified with neat PDMS. The obtained results from AFM and TEM analysis revealed that the modification of fumed silica with mixtures of PDMS-20/DEC allows obtaining narrow particle size distribution with uniform dispersity and an average particle size of 15–17 nm. The fumed silica nanoparticles chemically modified with mixtures of PDMS-x/DEC have potential applications such as nanofillers of various polymeric systems, thickeners in dispersing media, and additives in coatings.

## 1. Introduction

The hydrophobic nanodispersed silicas are widely used as thickeners in complex polar liquids based on epoxy, polyurethane, and vinylester resins. Additionally, such silicas improve the water resistance of moisture-sensitive formulations such as cosmetic preparations and flowability of powders. Also, chemically modified silicas are widely used as an anti-settling and anti-sagging agent of pigments, and in epoxy coatings, respectively. Organophilization of silica surfaces can be performed using various traditional kinds of modifying agents such as alkoxy-, halo-, aminosilanes and organosilazanes [1,2,3,4,5,6,7,8]. However, due to high reactivity and moisture sensitivity of above-mentioned modifying agents, purification is often critical for these hydrolyzable precursors. Poly(dimethylsiloxanes) provide a viable and environmentally benign alternative to the chemical functionalization of oxides as they are characterized by high carbon content, hydrophobic properties, thermal stability, chemical inertness, and they are noncorrosive reagents, and generate only water as a byproduct [9,10,11,12,13,14,15,16,17,18,19,20,21]. Despite this fact, the chemisorption of the simplest methylsiloxanes–hexamethyldisiloxane (H_3_C)_3_SiOSi(CH_3_)_3_ on fumed silica surfaces requires the use of very high temperatures (>350 °C) [19,22,23,24]. Additionally, the use of poly(dimethylsiloxanes), with high molecular weight for chemical modification of silica surfaces requires an even higher expenditure of energy (≥400 °C). One of the probable ways to increase the reactivity of an organosilicon polymer is partial depolymerization of high molecular poly(dimethylsiloxanes) followed by grafting formed oligomers (with terminated alkoxy groups) on silica surfaces. Partial depolymerization can be realized by different means: by thermal degradation (300–400 °C) or by treatment with toxic agents such as alkalis, sulfuric acids, thionyl chloride, amines, and mixtures of alkali (NaOH, KOH) with alcohols (methanol, ethanol) [25,26,27,28,29,30]. It should be noted, however, that the use of these catalysts is technologically complicated because of potential electrolyte presence in the obtained products, which are not desirable especially when using modified silicas as fillers in silicone cable rubbers and thickeners in insulating electrical greases.

**Scheme 1:**
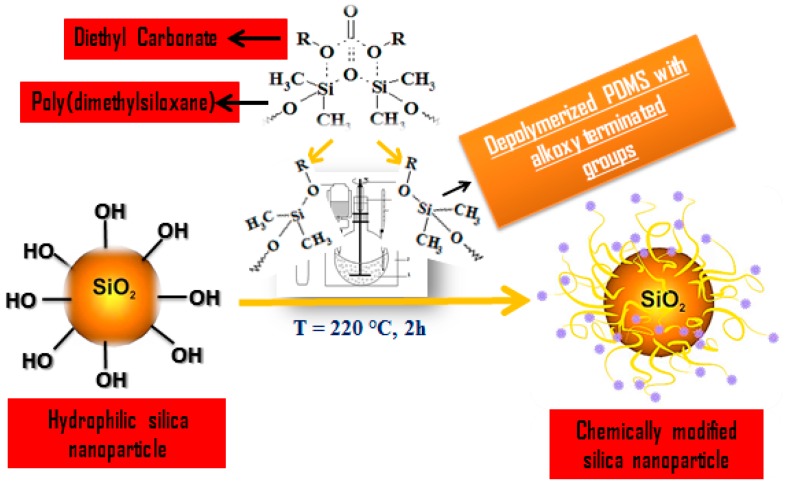
Synthesis of high disperse silicas with a high content of the grafted organic groups.

In our opinion, new, more favorable conditions come up when using poly(dimethylsiloxanes) for the chemical modification of silica surface in the presence of dialkyl carbonates (DMC or DEC) as initiators of siloxane bond splitting. Dialkyl carbonates are environmentally friendly reagents that meet all the requirements of green chemistry [31,32]. Japanese researchers showed that dimethyl or diethyl carbonate methanol solutions with the addition of alkali metal halogenides are effective agents in reactions of siloxane bond cleavage in the poly(dimethylsiloxanes) [33,34]. For the results of such reactions, alkoxysilanes (dimethyldimethoxysilane and trimethylmethoxysilane, see Scheme 1) were formed [34]:

Me_3_Si–[OSiMe_2_]*_n_*–Me + *n*(MeO)_2_CO → (*n* − 1)Me_2_Si(OMe)_2_ + 2Me_3_SiOMe + *n*CO_2_ (1)

A recent theoretical study [35] describes the role of the DMC in the Si–O bond cleavage in PDMSs as an activator of Si–O bond by coordination [O=C····O–Si and H_3_CO····Si–O], which largely decreases the electron density at the silicon center, promoting it easer cleavage.

In previous work [36], we determined that DMC without any addition of toxic reagents is an effective agent (initiator) in reactions of siloxane bond splitting in poly(dimethylsiloxanes) and on the silica surface sites. The chemisorption of depolymerized siloxane oligomers on silica surfaces occurred at a relatively low temperature (220 °C). Apart from the obvious technological advantages associated with the use of lower temperatures, this approach allowed us to obtain a high concentration of grafted organic groups in modified products (more than 10 wt %). Therefore, it is of interest to examine the effect of diethyl carbonate as initiator of siloxane bond cleavage in organosiloxanes and on surface silica sites when modifying the surface of SiO_2_ nanoparticles with the purpose to obtain hydrophobic products with uniform dispersity and high carbon content.

Therefore, the aim of the present study was the investigation of the features of DEC chemisorption on SiO_2_ surface and the chemisorption peculiarities of poly(dimethylsiloxanes) in the presence of DEC on silica surface. The main focus in this paper is lies on (1) the investigation of the reaction conditions for the covalent functionalization of fumed silicas using mixtures of PDMS-x/DEC, (2) the investigation of hydrophobicity of the obtained modified products and the study of their morphology and particle size (3) examining the influence of DEC presence in the modifier mixture on rheological behavior of modified silicas in industrial oil. All samples modified with mixtures of PDMS-x/DEC were compared with hydrophobic silicas modified with neat PDMS.

## 2. Materials and Methods

### 2.1. The Strategy of Silica Surface Modification

The strategy of the modification process of SiO_2_ nanoparticles with neat PDMS and PDMS-x/DEC mixtures (Scheme 1) was adopted from our previous work [36]. The chemisorption of PDMS on the fumed silica surface was performed at 220 °C for 2 h with or without addition of DEC (the volume ratio of PDMS-x/DEC was 1:1). Overall, the PDMS amount was 17% of silica weight. The modification of silica was carried out in a glass reactor with a glass stirrer (at rotational speed from 20 to 300 rpm). After loading with fumed silica, all the air volume in the reactor was replaced with nitrogen and the reactor was heated up to 220 °C. Further, the reactor filled with nitrogen was stopped and the modifying agent was added by spraying its aerosol through the nozzle. After heating, the mixture was cooled to room temperature. The removal of the physically adsorbed reactants was performed in a Soxhlet apparatus with *n*-hexane as a solvent at 68 °C for 1 h. Then, the washed samples were dried in the muffle furnace (ThermoLab SNOL 7,2/1100, Kyiv, Ukraine) at 80 °C for 2 h.

### 2.2. Reagents

Fumed silica (purity 99.87%, *S* = 260 m^2^/g) were supplied by the Pilot Plant of the Chuiko Institute of Surface Chemistry (Kalush, Ukraine). Commercial poly(dimethylsiloxanes) (code name: PDMS-100, linear, –CH_3_ terminated, viscosity 95–105 mm^2^/s molecular weight ~6000 Da and degree of polymerization 35–65), (code name: PDMS-50, linear, –CH_3_ terminated, viscosity 45–55 mm^2^/s, molecular weight ~3500 Da and degree of polymerization 23–28) and poly(dimethylsiloxane) (code name: PDMS-20, linear, –CH_3_ terminated, viscosity 18–22 mm^2^/s, molecular weight ~2000 Da and degree of polymerization 13–15). Diethyl carbonate containing ≥99.0 wt % of (C_2_H_5_O)_2_CO, *n*-hexane containing ≥99.0 wt % of C_6_H_6_ and hexamethyldisilazane (HMDS) was obtained by Sigma Aldrich (Hamburg, Germany).

### 2.3. Infrared Spectroscopy

In order to control the flow of surface reactions, IR spectra were recorded using a Specord M-80 spectrophotometer (Carl Zeiss, Jena, Germany) in the 4000–200 cm^−1^ wavenumber range. The silica samples were pressed into rectangular 28 × 8 mm plates of 25 mg weight.

The spectral investigations of the chemisorption processes of DEC on the silica surface were conducted in the quartz cuvette, which allowed us to perform thermal processing of the samples in vacuum conditions. The quartz cuvette contained infrared transparent glass made of fluorite. In the first stage, the sample of fumed silica was heated at 600 °C and pumped at a pressure of 10^−2^ mm·Hg in order to remove the interfacial and adsorbed water from the silica surface. At the second stage, the degassing of diethyl carbonate was performed by means of the freeze–pump–thaw degassing method. At the third stage, the prepared sample of dehydrated silica was brought into contact with saturated vapors of diethyl carbonate at different temperatures (200, 300 and 350 °C).

### 2.4. Contact Angles

Contact angle measurements were performed using a commercial Contact Angle Meter (GBX Scientific Instruments, Romans sur Isere, France) equipped with a temperature and humidity controlled measuring chamber and a digital camera (20 °C, relatively humidity is 50%). Firstly, hydrophobic powders were pressed into thin pellets (180 bars for 15 min). Then, a drop of deionized water was placed onto the surface and then plate was checked to determine the distribution of water on the surface. The contact angle was calculated using a computer program from a measurement of the width and height of the droplet. To obtain the averaged values, the measurements were performed for 6 water droplets put on each sample.

### 2.5. Elemental Analysis

The content of grafted organic groups in the synthesized samples was measured by Perkin-Elmer 2400 CHN-analyzer (Waltham, MA, USA). The anchored layer was oxidized to produce H_2_O and CO_2_ during the samples heating in the oxygen flow at 750 °C.

### 2.6. TEM and AFM

Transmission electron microscopy (TEM) images were recorded using FEI Tecnai G2 T20 X-TWIN, Hilllsboro, OR, USA. The powder samples were added to acetone (chromatographic grade) and sonicated. Then, a drop of the suspension was deposited onto a copper grid with a thin carbon film. After acetone evaporation, sample particles that remained on the film were studied with TEM. Additionally, the surface morphology and grain size distribution was analyzed using atomic force microscopy (AFM, Nanoscope V Digital Instruments, Boston, MA, USA, with a Tapping Mode technique). AFM data processing was performed using the SPIP program (version 5.0.6, Hørsholm, Denmark).

### 2.7. Rheology of Modified Silica Nanoparticles

Modified silica suspensions were prepared at concentrations of solid phase 5 wt % in industrial oil (I-40A) at room temperature (25 °C). Rheological properties of the silica suspensions were studied with a rotational viscosimeter Reotest RV2.1 (“Mettingen” firm, Mettingen, Germany,) equipped with a cylindrical system at the shear rates (*γ*) from 9 to 1312.2 s^−1^. The industrial oil was employed as a dispersion media as hydrophobic silica has a wide range of applications as thickeners for nonpolar fluids.

## 3. Results and Discussion

### 3.1. Infrared Spectroscopy Analysis Results

**Scheme 2:**
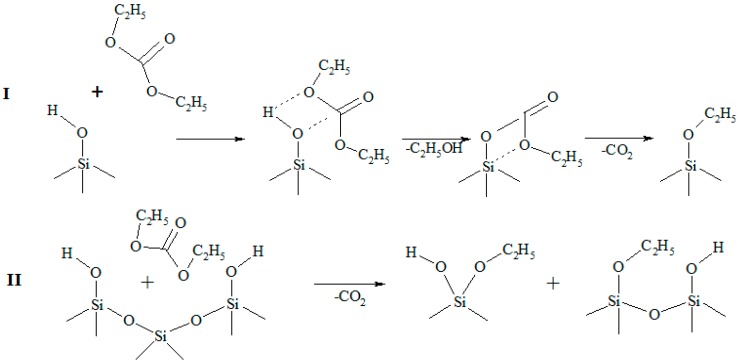
(**I**) Attack by diethyl carbonate (DEC) of silica silanol group; (**II**) attack by DEC of silica Si–O bond.

We have already shown [36] that chemical interaction of dimethyl carbonate with sites of the dehydrated silica surface takes place at a temperature of 200 °C and higher. Chemisorption processes involve both structural silanol groups and siloxane bridges on the surface. In this paper, we investigated the peculiarities of diethyl carbonate chemisorption on the dehydrated silica surface. At the first stage, both features of the interaction of diethyl carbonate with structural silanol groups on the silica surface (see Scheme 2I) and possibilities of the cleavage of siloxane bonds located directly on the dehydrated silica surface were investigated (see Scheme 2II).

**Figure 1:**
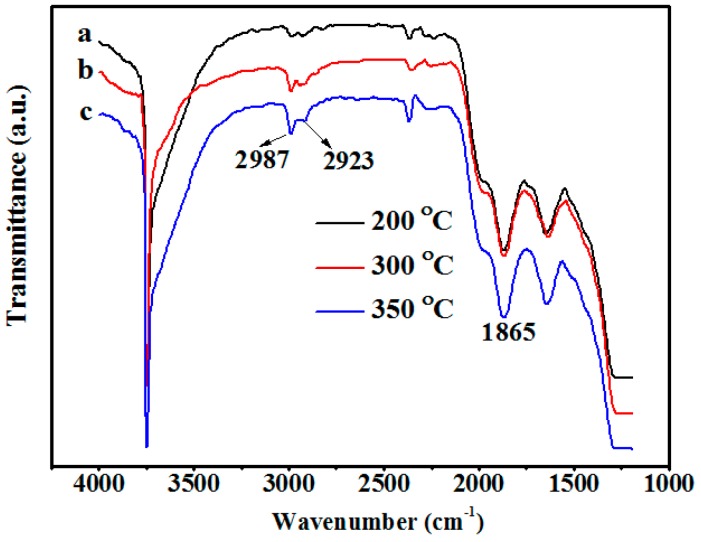
IR spectra of dehydrated silica after interaction with saturated vapors of diethyl carbonate at (a) 200 °C; (b) 300 °C; (c) 350 °C and subsequent vacuum treatment of the samples at the same temperatures.

It was found that the chemical interaction of DEC ((H_5_C_2_O)_2_CO) with the surface sites of dehydrated silica takes place when the temperature increases up to 200 °C (Figure 1a).

**Figure 2:**
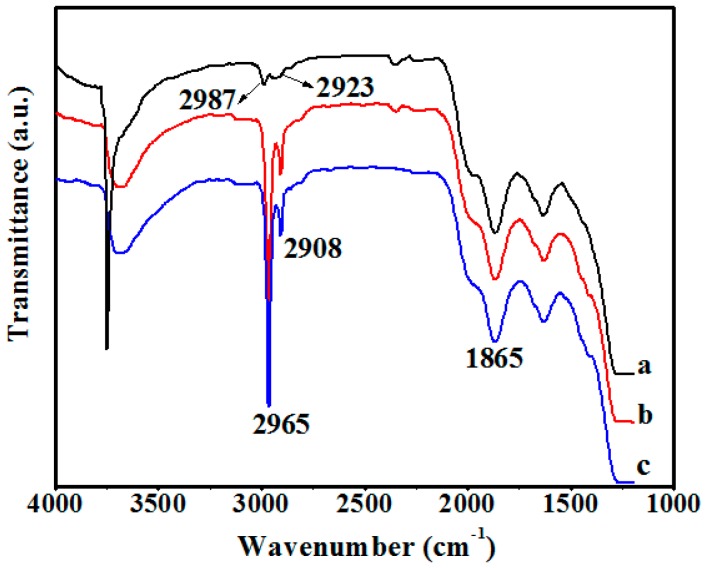
IR spectra of fumed silica after (a) reaction with vapors of diethyl carbonate; (b) reaction with vapors of hexamethyldisilazane at 120 °C subsequent vacuum treatment of surface at 300 °C; (c) interaction with vapors of the diethyl carbonate and vacuum treatment at 300 °C.

When the temperature rises further, the increase in the intensity of bands corresponding to the stretching vibrations C–H at 2987 and 2923 cm^−1^ in grafted ethoxy groups are observed. It should be noted that the concentration of grafted ethoxy groups (–OC_2_H_5_) increases and full participation of silanol groups (O–H) in the chemical reaction with diethyl carbonate under these conditions is not observed. Therefore, it was logical to assume that the chemisorption of (H_5_C_2_O)_2_CO proceeds via the siloxane bond cleavage on the silica surface. To test this assumption and to better understand the processes of chemisorption of diethyl carbonate, the silanol groups were removed from the silica surface by being substituted with trimethylsilyl groups as a result of the reaction with hexamethyldisilazane (Figure 2b).

**Scheme 3:**
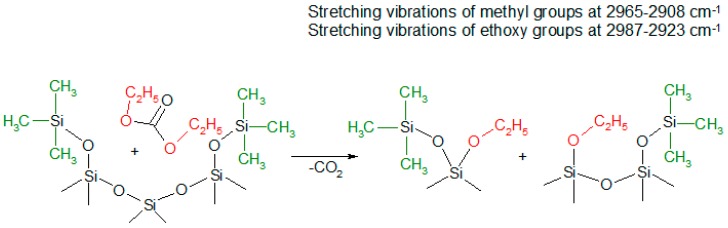
Scheme of interaction of DEC with silica surface which were treated with HMDS at 300 °C.

From Figure 2b, we can see that silanol groups are not observed on the spectra with simultaneous appearance of the stretching vibrations of methyl groups (2908–2965 cm^−1^). However, after the contact with vapors of (H_5_C_2_O)_2_CO at 300 °C and the subsequent vacuum treatment of the surface, the band with maximum at 2923 and 2987 cm^−1^ is very hard to distinguish from the valence vibrations of methyl groups as they absorb at the same frequency ranges (Figure 2c, see Scheme 3).

In summary, we can say that DEC reacts with silanol groups on the silica surface forming the grafted ethoxy groups which can act as an additional reactive center for chemisorption of organosiloxanes. However, it is very hard to say that DEC reacts with the Si–O bond directly at the SiO_2_ surface, but we can assume that this reaction may occur due to the above-mentioned context.

**Figure 3:**
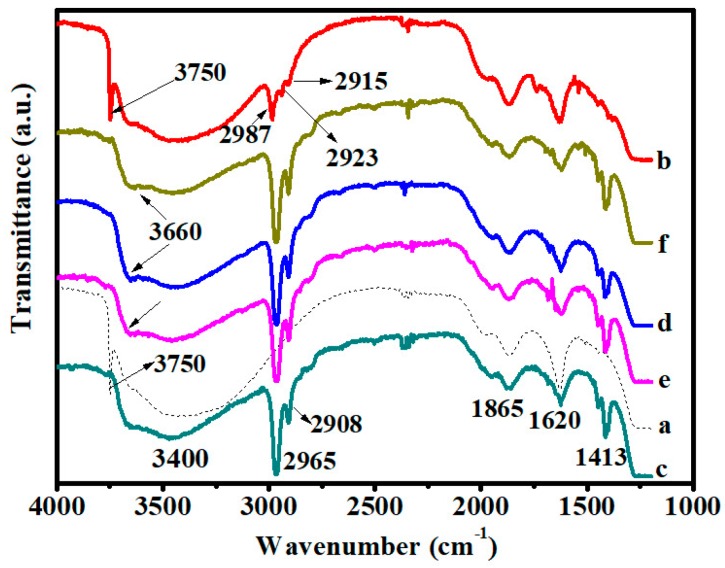
IR spectra of (a) neat fumed silica; (b) fumed silica modified with neat DEC; and (c) silica modified with neat poly(dimethylsiloxanes) (PDMS)-50; (d) PDMS-20 and ((e,f) respectively) their mixtures with DEC at 220 °C for 2 h.

**Figure 4:**
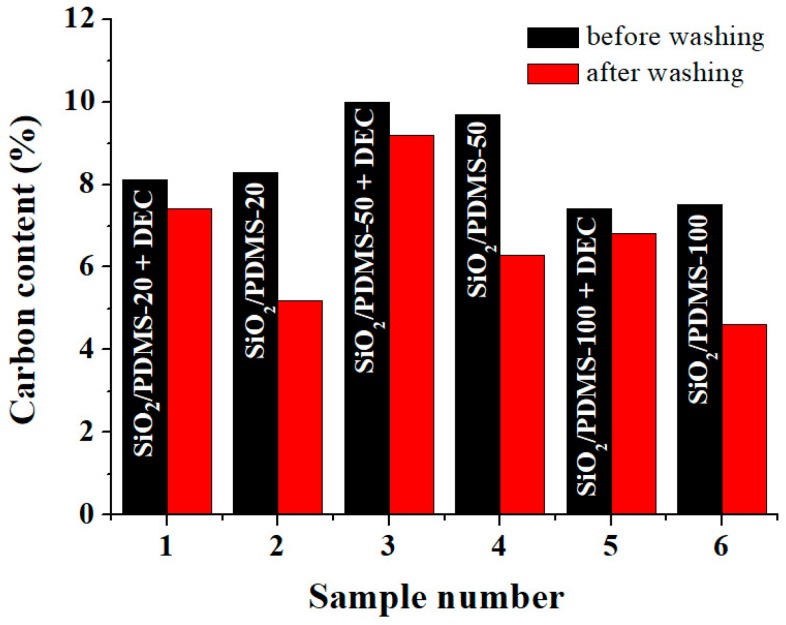
Histogram of carbon content for modified silicas: **1**—SiO_2_/PDMS-20 + DEC, **2**—SiO_2_/PDMS-20, **3**—SiO_2_/PDMS-50 + DEC, **4**—SiO_2_/PDMS-50, **5**—SiO_2_/PDMS-100 + DEC, **6**—SiO_2_/PDMS-100.

Changes in surface structure of modified silicas are clearly visible in the IR spectra (Figure 3). Figure 3 shows the IR spectra of neat SiO_2_ nanoparticles (Figure 3a), silica modified with neat DEC (Figure 3b) and composites prepared by modification of SiO_2_ surface with neat poly(dimethylsiloxanes) (PDMS-20 or PDMS-50) and their mixture with DEC (Figure 3c–f). In the spectrum of the silica which was modified only with diethyl carbonate (Figure 3b), we can see the presence of valence vibrations of C–H bond in ethoxy groups at 2987–2908 cm^−1^, the peak attributed to absorbed water at 3700–3400 cm^−1^ and 1620 cm^−1^ and also vibration of silanol groups at 3750 cm^−1^. The spectrum of neat fumed silica (Figure 3a) is characterized by the presence of valence vibration of silanol groups at 3750 cm^−1^ and the valence and deformation vibration of absorbed water at 3700–3400 cm^−1^ and 1620 cm^−1^, respectively. Intensive band at 2965 cm^−1^ (asymmetric C–H vibrations in methyl group) and accompanying band at 2908 cm^−1^ (symmetric C–H vibrations) in IR spectra of modified silicas samples indicate high concentration of grafted methylsiloxane which is in accordance with the data on carbon content (Figure 4, discussed in Section 3.2) demonstrating high yield of methylsiloxane grafting.

However, the free silanol band at 3750 cm^−1^ is no longer detected. The signal of the free silanol groups disappeared completely from the spectra of modified silicas confirms the passage of the reaction between the silica surface and modifier agents. Note that the intensity of a silanol band at 3660 cm^−1^ changes much less than the 3750 cm^−1^ free silanol band. The former is attributed to silanols that are less accessible [37] to siloxane molecules during the surface modification. In addition, we can see that the intensity of band at 3700–3400 and 1620 cm^−1^ corresponding to adsorbed water is detectable on the surface of all modified samples, but the intensity of this peak slightly decreases for silicas modified by mixtures of PDMS-x/DEC (Figure 3e,f). This could be explained by that fact that siloxane oligomers which were formed as the result of DEC and PDMS interaction have reacted with surface silanols more intensively than neat PDMS macromolecules.

At any rate, we still can see that individual molecules or clusters of water remained in the adsorption layer of oxide composites despite the presence of hydrophobic PDMS. The presence of adsorbed water clusters can be explained by the textural features of the nanosilica powders. Water molecules are much smaller than the cross-section of PDMS. Therefore, water can penetrate into narrow nanovoids in the contact zones between adjacent nanoparticles in aggregates but PDMS molecules cannot penetrate into these voids.

### 3.2. Elemental Analysis Results

From the elemental analysis data (Figure 4), one can see that the highest concentration of grafted organic groups in modified samples (>9 wt % of carbon content) is achieved by using of PDMS-50/DEC mixture for the modification.

In addition, we can see that the carbon content is not practically changed after the treatment in organic solution for silicas modified with mixtures of PDMS-x/DEC, which indicates that organosiloxanes are chemically bonded with the silica surface sites. For samples modified in the absence of DEC after the treatment in the same conditions, the carbon content decreases about 3 wt %, which is evidence of partial desorption of the grafted organic layer into the solvent. The obtained elemental analysis data is in good agreement with data of rheology, discussed later.

**Figure 5:**
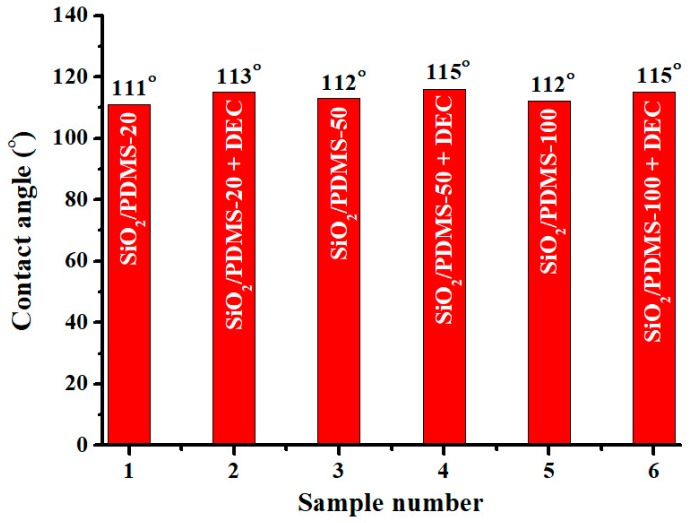
Contact angles of wetting of modified silicas.

The value of the contact angle of wetting (Figure 5) for such powders was measured to be about 115–116° which indicates the hydrophobic surface. Samples synthesized in the presence of DEC were characterized by slightly higher values of the contact angle of wetting.

### 3.3. Rheology Analysis Results

**Figure 6:**
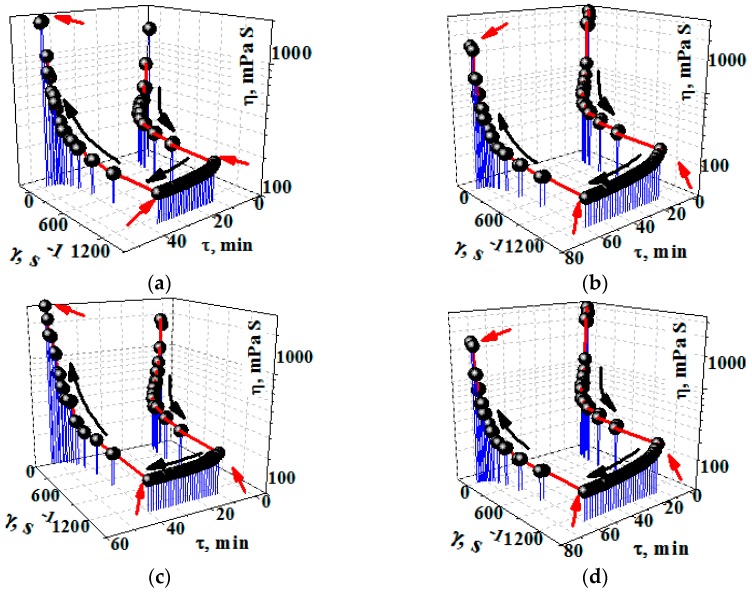
Dependence of effective viscosity on the shear rate over time for hydrophobic silica modified with (**a**) mixture of PDMS-20/DEC; (**b**) neat PDMS-20; (**c**) mixture of PDMS-50/DEC; (**d**) neat PDMS-50 in industrial oil I-40A with the concentration of solid phase 5 wt %.

Modified disperse silicas are widely used as rheological additives, which are key ingredients in paints, coatings and inks as they control the precise properties and characteristics of fluid products. Therefore, the rheological properties in industrial oil of modified silicas were examined (Figure 6a–d).

Due to electrostatic interactions, silicas modified with neat organosiloxanes (PDMS-20 or PDMS-50) and their mixture of DEC disperse in coating formulations and create a three-dimensional network, which results in a viscosity increase in the formulation. Under shear force, the three-dimensional network collapses and agglomerates of modified silica move freely through the liquid, and as a result viscosity decreases (Figure 6a–d). After the shear force weakens, the modified silica network spontaneously reforms and the viscosity increases again (Figure 6a–d). This fully reversible network is the key to success for rheology control with modified silicas. Figure 6b,d reveals that dispersion of modified silicas with neat PDMS-20 or PDMS-50 are characterized by a low structure recovery of the interparticle bonds by reducing a shear rate, i.e., with mechanical influence on the thickened suspensions a partial destruction of the structure is observed. In contrast, silica dispersions modified with mixtures of PDMS-20 or PDMS-50 in the presence of DEC (Figure 6a,c) are clearly characterized by a fully reversible inner structure and thixotropic behavior as the viscosity increases to initial values. In addition to this, silicas modified with mixtures of PDMS-x/DEC require shorter time for the structure recovery after the shear force was applied which can be explained by the formation of stable bonds between silica and organosilicone oligomer. On the contrary, for SiO_2_ which was modified with neat PDMS-20 or PDMS-50 the definite part of oligomer is physically adsorbed on the surface and a partial destruction of the colloidal structure is observed under the mechanical action as the viscosity does not increases to initial values.

### 3.4. AFM and TEM Analysis Results

**Figure 7:**
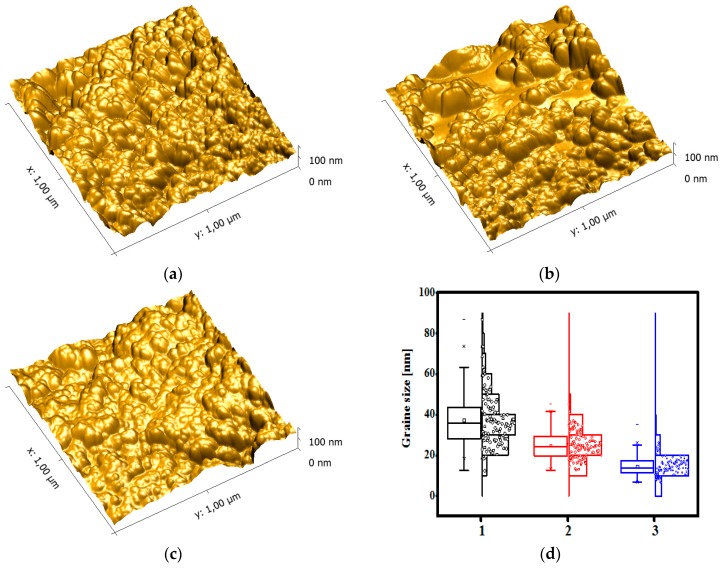
Atomic force microscopy (AFM) images of (**a**) initial SiO_2_ nanoparticles; (**b**) SiO_2_ modified with neat PDMS-20; (**c**) SiO_2_ modified with mixtures of PDMS-20/DEC and (**d**) their histogram: 1—initial SiO_2_ nanoparticles, 2—SiO_2_ modified with neat PDMS-20, 3—SiO_2_ modified with mixtures of PDMS-20/DEC.

Figure 7 illustrates AFM images and the histogram (depicting the nanoparticle size distribution) of initial SiO_2_ nanoparticles and fumed silica modified with neat PDMS-20 and its mixture with DEC.

Notice that particles of modified silicas are smaller (Figure 7b–d) than those of bare SiO_2_ (Figure 7a,d). This occurs due to the heating of modified samples during synthesis and thus the formation of smaller nanoparticles. The silica modified with mixture of PDMS-20/DEC (Figure 7c,d) characterized by homogenous particle size distribution has an average grain size of 15–17 nm. This is easy to explain assuming that firstly, DEC can act as a blocker and a separator that prevents the aggregation of modified SiO_2_ nanoparticles and thus hinders any further growth of SiO_2_ particles; secondly, the chemisorption of depolymerized poly(dimethylsiloxane) accompanied by the grafting of shorter polymer chains on the SiO_2_ surface which improves the distribution of the depolymerized PDMS-20 on the SiO_2_ surface; thirdly, the short polymeric grafted chains can control the nanoparticle aggregation by steric repulsions.

In contrast, the modification of SiO_2_ with neat PDMS-20 (Figure 7b,d) leads to an increase in particle size up to 25–30 nm. This can be explained by the fact that the reaction of neat polymers with SiO_2_ surface can run through the island-like polymer distribution onto the silica surface (as the chains of neat polymers is very long and it is difficult to spread them out over the surface) when bonded molecules favor other molecules to be bound nearby which may cause the aggregation of SiO_2_ nanoparticles.

**Figure 8:**
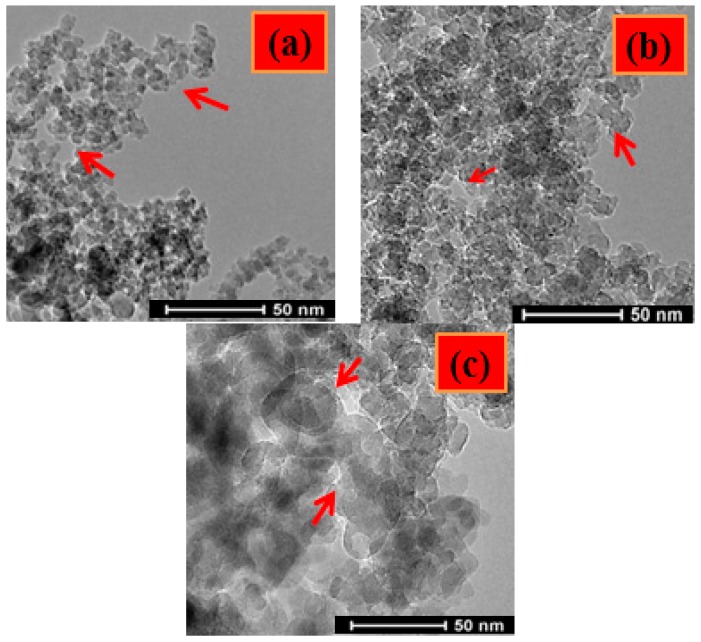
Transmission electron microscopy (TEM) images of (**a**) bare fumed silica; (**b**) silicas modified with mixtures of PDMS-20/DEC and (**c**) neat PDMS-20.

The morphology of neat silica and modified silicas were also analyzed using transmission electron microscopy (Figure 8). Initial fumed silica in this study is composed of nonporous nanoparticles with a true density of amorphous nanosilica *ρ*_o_ = 2.2 g/cm^3^ forming aggregates (Figure 8a) and agglomerates of aggregates. The particles obtained using for the modification of SiO_2_ mixtures of PDMS-20/DEC exhibit a lesser degree of aggregation than those obtained via modification of SiO_2_ surfaces by neat PDMS-20. SiO_2_, grafted with depolymerized PDMS-20 shows no sign of a visible coating (Figure 8b). Therefore, it is assumed that the functionalization took place via the attachment of a monolayer of the respective PDMS-20/DEC mixture on the surface of SiO_2_. On the contrary, it is possible to depict some part of polymer on a SiO_2_ surface which was modified with neat PDMS-20 (Figure 8c). Voids between primary nanoparticles in the secondary structures are responsible for the textural porosity of the silica powders and they are present for both bare silica and for modified samples [37,38,39,40,41]. In general, the hydrophobization of the silica surface with siloxanes to form a mosaic coverage with methyl groups affects the particulate morphology.

## 4. Conclusions

In the present work, the features of DEC chemisorption on a fumed silica surface were examined, and it was found that the chemical interaction of diethyl carbonate with sites of the dehydrated silica surface takes place at a temperature of 200 °C and higher, and that chemisorption processes involve structural silanol groups.

The reaction of neat poly(dimethylsiloxanes) and their mixture with DEC with fumed silica were investigated. It was established that the reaction of siloxane oligomers with free silanol groups on silica surface in the presence of diethyl carbonate takes place at relatively low temperatures (220 °C) with the formation of stable hydrophobic coating (exhibiting contact angles 113–115°) in which the carbon content is higher than 8 wt %. The modification of SiO_2_ surface with organosiloxanes without diethyl carbonate leads to the formation of patches of an absorbed organic layer which is easily desorbed in polar media.

The results of TEM and AFM studies revealed that modified SiO_2_ nanoparticles with mixtures of PDMS-20/DEC exhibit a lesser degree of aggregation than those obtained using neat PDMS-20 for modification. The average particle size of the modified SiO_2_ with mixtures of PDMS-20/DEC is determined to be 15–17 nm. The research of rheological properties of modified silicas in industrial oil has shown that the dispersion of silicas, which were modified with mixtures of PDMS-x/DEC exhibits thixotropic behavior and is characterized by a fully reversible network and a short recovering time. In contrast, the partially irreversible destruction of the structure is observed for SiO_2_ modified with neat PDMS.

To summarize, we have developed a highly efficient (carbon content >8 wt %), relatively low temperature (220 °C) and environmentally friendly grafting approach for surface functionalization of silicas by using mixtures of PDMS-x/DEC. Such silicas, with a covalently attached hydrophobic layer, can find their applications as additives in coatings and paints, fillers in various polymeric systems and thickeners in optic cable gels.
